# Potential impact of climatic factors on the distribution of *Graphium sarpedon* in China

**DOI:** 10.1002/ece3.10858

**Published:** 2024-02-06

**Authors:** Wenkai Liao, Zhihang Zhuo, Qianqian Qian, Dan Hu, Danping Xu

**Affiliations:** ^1^ College of Life Science China West Normal University Nanchong China

**Keywords:** ArcGIS, climatic conditions, environmental variables, *Graphium sarpedon*, MaxEnt model, potential distribution area

## Abstract

*Graphium sarpedon* is a significant foliar pest of Laurel plants in China. In this study, the MaxEnt model was used to investigate the distribution of *G. sarpedon* and predict its potential distribution areas in China in the future (2050s and 2090s) based on three Shared Socioeconomic Pathways (SSP1‐2.6, SSP2‐4.5, and SSP5‐8.5), and key environmental variables affecting its distribution were identified. The results showed that under the current climatic conditions, the suitable distribution areas of *G. sarpedon* were 92.17°–134.96° E and 18.04°–33.61° N, including Yangtze Plain (Middle and Lower), Pearl River Delta, Yangtze River Delta, and Lingnan areas. Under the future climate conditions, the total suitable distribution area of *G. sarpedon* decreased, but the area of medium suitable area increased. The study identified 11 key environmental variables affecting the distribution of *G. sarpedon*, the most critical of which was Precipitation of Warmest Quarter (bio18) and precipitation in April, May, June, and September (prec4, prec5, prec6, and prec9). This study is beneficial for monitoring and preventing the possible changes of *G. sarpedon* and provides theoretical references for its prevention and control.

## Graphical abstract

In this study, we analyzed the suitable distribution areas of *Graphium sarpedon* under current and future climate change (SSP1‐2.6, SSP2‐4.5, and SSP5‐8.5) for two periods (the 2050s and 2090s) using the MaxEnt model. The analysis results showed that the high suitable areas are mainly distributed in southern Guangdong, southwestern Guangxi, western Taiwan, Hong Kong, and Zhejiang. Under different future climatic conditions, some of the high, medium, and low suitability zones for *G. sarpedon* increased and some decreased, but the mass center did not migrate significantly.

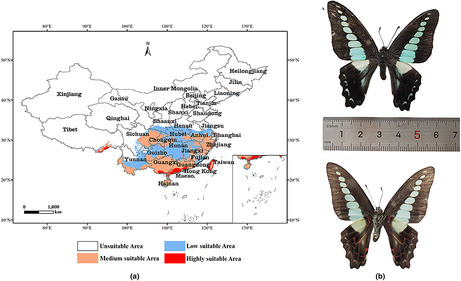

## 1 INTRODUCTION

*Graphium sarpedon*, a major pest of Lauraceae plants, belongs to the Papilionidae family of Lepidoptera. It is primarily found in areas with tropical and subtropical monsoon climates in China, such as Guangdong, Guangxi, Hainan, Hong Kong, and Taiwan (Xiao, 2020). In recent years, *G. sarpedon* has expanded its range and infestation has become an increasing problem. The main host plant of *G. sarpedon* is *Cinnamomum camphora* (Zhang, 2016), which is a beautiful, evergreen tree and an important garden tree in southern China, and the larvae of *G. sarpedon* will bite the new leaves of *C. camphora*, and in severe cases, only the midvein of the leaves is left, and the branches only have old leaves, causing the crown to become bald. In severe cases, the larvae eat only the midrib of the leaves, leaving only some old leaves on the branches, resulting in a bare crown, affecting the growth of trees, and causing great damage to the ecological environment of parks and neighborhoods (Wu, 2018).

Host plants have a strong influence on the insect's range of activity, and this is also true for *G. sarpedon*. *G. sarpedon* mainly parasitizes camphoraceous plants, and it has been found that the foliar volatiles of camphoraceous plants show activity against *G. sarpedon* in both electrophysiological and behavioral tests, and with the widespread cultivation of such plants, *G. sarpedon*'s range of suitability may have been altered (Li et al., 2010). Climate change affects a wide range of ecological phenomena and processes, and many studies have shown that changes in species distributions, morphological traits, and biophysical climates can be significantly affected by climate change (Lenoir et al., 2008; Parmesan & Yohe, 2003; Thomas et al., 2004). For butterflies, changes in climate can affect flight capacity, with warmer temperatures leading to a significant increase in the number and duration of flights associated with territorial behavior, suggesting that high temperatures are particularly important for sustaining high‐energy flights involved in territorial defense and mate interception, which may affect the expansion potential of butterfly populations (Hayes et al., 2019). The sudden increase in the concentration of greenhouse gases in the atmosphere in recent years has led to dramatic changes in the Earth's climate environment, which will bring about drastic changes in the distribution of species (Shi et al., 2023). Understanding changes in the suitable distribution area of *G. sarpedon* under future climate change scenarios and taking targeted preventive measures are important for future *C. camphora* pest control.

To predict species distributions based on the relationship between species occurrence and variables, species distribution models (SDMs) are widely used as inference tools (Coro et al., 2018). MaxEnt is one of the most commonly used SDMs for species distribution modeling, which is based on species occurrence records and modeling of environmental variables (Phillips et al., 2006). Compared with other models, MaxEnt has higher simulation accuracy and is characterized by short run time, simple operation, stable run results, and low model sample size requirements (Li et al., 2013; Zhang, 2015). It has been widely recognized and used to predict the fitness simulation of various pests and diseases (Bosso et al., 2016; Han et al., 2015; Qi et al., 2012; Wenjuan & Lin, 2009).

Therefore, we predicted future fitness zones of *G. sarpedon* using the MaxEnt model to understand the importance of climatic conditions on the distribution of *G. sarpedon*. These studies can help to interpret and predict potential distribution trends of *G. sarpedon*, especially if climate change continues. In addition, the potential ranges of *G. sarpedon* were analyzed with the aim of providing a theoretical basis for understanding the ecological niche of *G. sarpedon* and contributing to the development of effective control measures.

## 2 MATERIALS AND METHODS

### 2.1 Species presence records

A total of 469 worldwide localities of *G. sarpedon* were used as occurrence data, which were obtained through field studies and collected from the Global Biodiversity Information Facility (GBIF) website (https://www.gbif.org/). The latitude and longitude of the localities were determined using a global positioning system during the field study. The distribution point data for *G. sarpedon* were saved as a CSV (comma‐separated values) grid in Excel format. The fields included serial number, location, longitude, latitude, and references. All species occurrences in this study were filtered to remove location information that did not provide accurate latitude and longitude as well as duplicates in the database. Distribution point data for *G. sarpedon* were saved in Excel in “CSV” (comma‐separated value) format. To avoid overfitting in the MaxEnt model, the ENMTools version 1.0.4 of the R platform was used in this paper to spatially filter the data according to longitude, keeping only one point in each grid cell (1 × 1 km) (Cao et al., 2011; Li et al., 2020).

### 2.2 Environmental parameters

The environmental variables involved in this study were 24 environmental variables, including 19 bioclimatic variables (bio1–bio19), one climate indicator (Prec), and one topographic data (aspect) (Table 1). The environmental variable data were all downloaded from WorldClim (https://www.worldclim.org/) with “current period” of 1970–2000 (Jiang et al., 2019). Additionally, future climate data for the 2050s (2041–2060) and 2090s (2081–2100) were obtained from the CCAFS (Climate Change, Agriculture, and Food Security) website. Climate data were defined from 1970 to 2000, and three Shared Socioeconomic Pathways (SSPs) of SSP1‐2.6, SSP2‐4.5, and SSP5‐8.5 were selected to simulate the future distribution of this species, representing minimum, medium, and maximum greenhouse gas emission scenarios, respectively (Wang et al., 2018). The spatial resolution of the data used in this study was 2.5 arcminute (approximately 4.5 km^2^). In order to improve the accuracy of the model, it is necessary to exclude environmental variables with small contributions (Banerjee et al., 2019; Jiang et al., 2019; Zeng et al., 2016). Thus, we used the jackknife test in MaxEnt v3.4.4 (https://biodiversityinformatics.amnh.org/open_source/maxent/, RRID:SCR_021830) to determine the degree of contribution of each environmental variable to model construction and eliminate the variables with small contributions (Zhang et al., 2015). In order to improve the accuracy of the model, variance inflation factor (VIF) analysis was performed on all environmental variables (Phillips et al., 2006). Finally, environmental variables with VIF < 100 were selected for the prediction model.

**TABLE 1:** A list of environmental variables with abbreviations and variable meaning.

Environment variables	Abbreviation	Variable meaning
Annual mean temperature	bio1	Reflect the characteristics of temperature
Mean diurnal range (mean of monthly) (max temp–min temp)	bio2	
Isothermality (bio2/bio7) (* 100)	bio3	
Temperature seasonality (standard deviation * 100)	bio4	
Max temperature of Warmest Month	bio5	
Min temperature of Coldest Month	bio6	
Temperature annual range (bio5–bio6)	bio7	
Mean temperature of Wettest Quarter	bio8	
Mean temperature of Driest Quarter	bio9	
Mean temperature of Warmest Quarter	bio10	
Mean temperature of Coldest Quarter	bio11	
Annual precipitation	bio12	Reflect the characteristics of precipitation
Precipitation of Wettest Month	bio13	
Precipitation of Driest Month	bio14	
Precipitation seasonality (coefficient of variation)	bio15	
Precipitation of Wettest Quarter	bio16	
Precipitation of Driest Quarter	bio17	
Precipitation of Warmest Quarter	bio18	
Precipitation of Coldest Quarter	bio19	
Precipitation	Prec	Reflect the characteristics of precipitation
Aspect	Aspect	Reflect the direction of the slope

### 2.3 MaxEnt modeling construction

In this study, MaxEnt v3.4.4 was used to build the prediction model. The occurrence data and environmental variable data of *G. sarpedon* were imported into the software (Wan et al., 2019), and 75% of the occurrence data were randomly selected as the training set to establish the model, while the remaining 25% of the distribution points of *G. sarpedon* were used as the test set to validate the model, and it is repeated 10 times. The importance of each environmental factor in the occurrence of *G. sarpedon* was analyzed using the jackknife method. An environmental variable response curve was also created, and default values were selected for the rest of the model parameters (Xu et al., 2022).

The receiver operating characteristic (ROC) curve analysis was used to evaluate the accuracy of each prediction model. The area under the ROC curve (AUC) was used as the evaluation criterion as it is not influenced by the threshold or other factors and has been widely used to evaluate model accuracy (Vanagas, 2004). The MaxEnt model automatically generated the ROC curve and the corresponding AUC value. The evaluation criteria for AUC were as follows: 0.5 ≤ AUC < 0.6 indicated that the prediction results failed; 0.6 ≤ AUC < 0.7 indicates poor predictions; 0.7 ≤ AUC < 0.8 indicated that the prediction results were available; 0.8 ≤ AUC < 0.9 indicated that the prediction results were good; and AUC > 0.9 indicated that the prediction results were excellent (Araujo et al., 2005; Swets, 1988) (Table S1). The closer the AUC value was to 1.0, the higher the accuracy of the model, and the optimal predictive model could be identified.

The MaxEnt model and ArcGIS for Desktop Basic 3.3 (http://www.esri.com/software/arcgis/arcgis‐for‐desktop, RRID:SCR_011081) were used to generate a probability distribution map of *G. sarpedon* in China. To classify the levels of distribution values and the corresponding distribution ranges, a “reclassification” function was used, based on the probability classification methods suggested in the report of the Intergovernmental Panel on Climate Change (IPCC). The specific criteria for classification were as follows: *p* < .05 (non‐suitable area); 0.05 ≤ *p* < .33 (low suitable area); 0.33 ≤ *p* < .66 (medium suitable area); and *p* ≥ .66 (highly suitable area) (Wang et al., 2023). White, blue, orange, and red colors indicate unsuitable areas, low suitable areas, medium suitable areas, and high suitable areas, respectively.

## 3 RESULTS

### 3.1 Model validation

The ROC curve and AUC value of the rebuilt model are presented in Figure S1, which shows that the AUC value of the geographic distribution model of *G. sarpedon* in China based on all environmental variables and dominant environmental variables was 0.977 > 0.9. According to the classification criteria in Table S2, the accuracy of the model was classified as “excellent.” These results demonstrate that the model is reliable and can be used to predict the potential distribution of *G. sarpedon*.

### 3.2 Potential distribution of *Graphium sarpedon* in the current period

**FIGURE 1:**
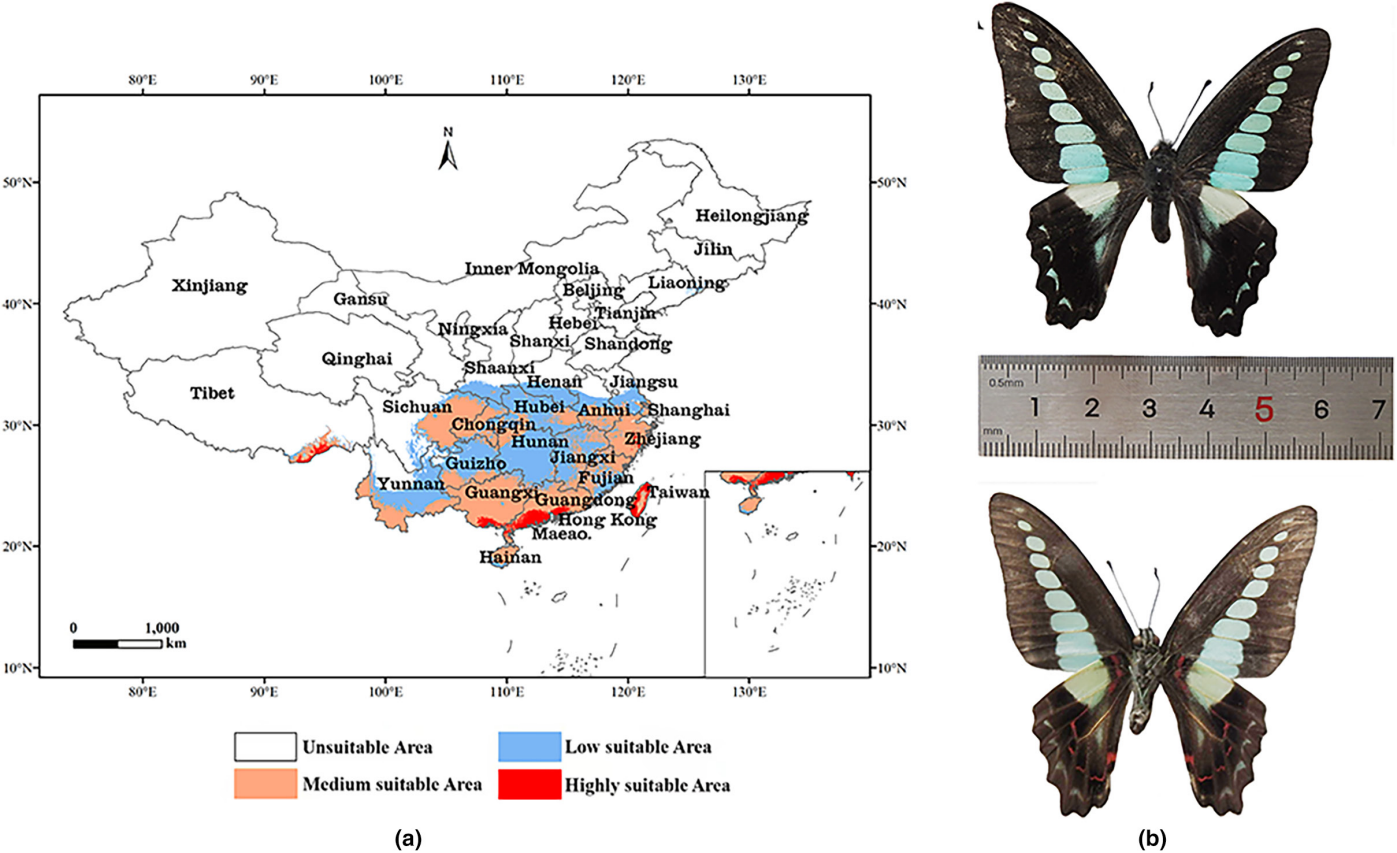
(a) Potential distribution areas of *Graphium sarpedon* in the current period. Unsuitable, low suitable, medium suitable, and highly suitable areas represent the occurrence probability of 0–0.05, 0.05–0.33, 0.33–0.66, and 0.66–1.00, respectively. (b) The dorsal and ventral surfaces of adult *G. sarpedon*.

The potential distribution regions of *G. sarpedon* in the current period are presented in Figure 1. The analysis predicted that the suitable range of *G. sarpedon* under contemporary climatic conditions was located between 92.17°–134.96° E and 18.04°–33.61° N. The suitable areas have been mainly concentrated in regions with tropical and subtropical monsoon climates, such as the Yangtze Plain (Middle and Lower), Pearl River Delta, Yangtze River Delta, and Lingnan areas (South of Nanling, China).

Based on the information presented in Table S3, the total suitable area for *G. sarpedon* in China is currently estimated to be 206.67 × 10^4^ km^2^, which represents approximately 21.52% of the total land area of China. The medium suitable area has the largest coverage of 101.24 × 10^4^ km^2^, accounting for 10.55% of China's land area. The low suitable area covers 97.75 × 10^4^ km^2^, accounting for 10.18% of China's total land area, while the highly suitable area has the smallest coverage of 7.68 × 10^4^ km^2^, accounting for 0.80% of China's land area.

The predicted areas for different suitability levels in each province are listed in Figure 1 and Table S3. The results showed that *G. sarpedon* was potentially distributed in 24 provinces in China, accounting for 70.59% of the total provincial administrative regions. Guangdong had the highest highly suitable area of 3.81 × 10^4^ km^2^. Guangxi and Taiwan also have large areas of highly suitable areas, 0.96 × 10^4^ and 1.57 × 10^4^ km^2^, respectively. Most of the highly suitable areas were located in the coastal areas, such as Guangxi, Guangdong, Hainan, Taiwan, Hong Kong, and Zhejiang provinces. In addition to this, Tibet also has a small distribution of highly suitable areas (0.99 × 10^4^ km^2^). Medium suitable areas are distributed around high suitable areas, including Guangxi, Guangdong, Taiwan, Zhejiang, Tibet, Fujian, Hong Kong, and other provinces. There are also large areas of medium suitable areas in Sichuan, Hainan, Yunnan, Anhui, and other provinces. Low suitability areas are mainly distributed in Hubei, Hunan, Guizhou, and Yunnan provinces, but also in Sichuan, Chongqing, Henan, Anhui, and Jiangsu provinces. It is worth noting that a small number of unsuitable areas are also distributed in Liaoning and Jilin.

### 3.3 Potential distribution of *Graphium sarpedon* in future period

**FIGURE 2:**
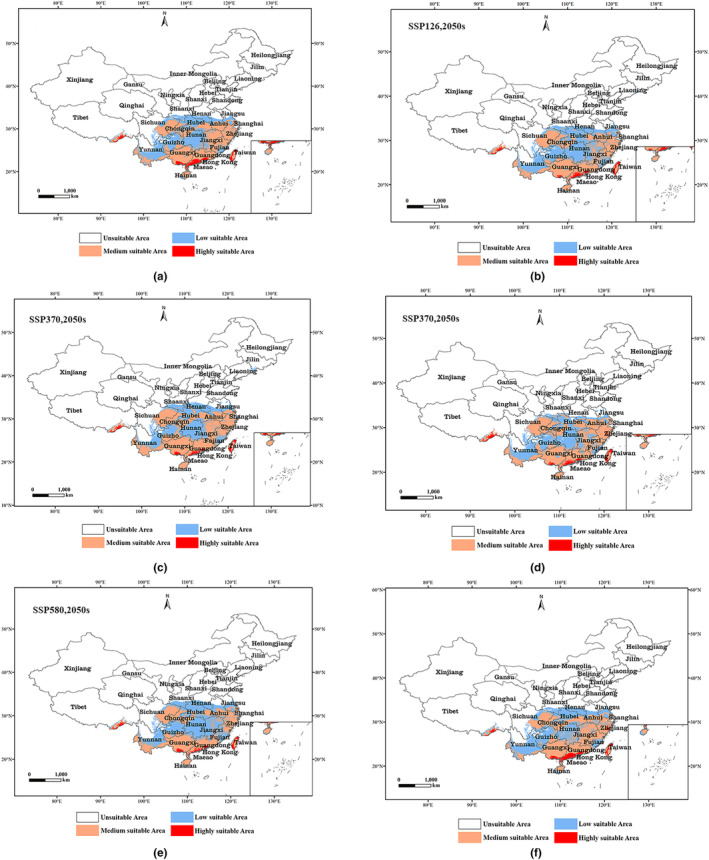
Comparison of potential distribution areas of *Graphium sarpedon* between 2050s and 2090s in China. (a) SSP1‐2.6 (2050s); (b) SSP1‐2.6 (2090s); (c) SSP2‐4.5 (2050s); (d) SSP2‐4.5 (2090s); (e) SSP5‐8.5 (2050s); (f) SSP5‐8.5 (2090s).

Figure 2 illustrates the potential distributions of *G. sarpedon* under three climate change scenarios (SSP1‐2.6, SSP2‐4.5, and SSP5‐8.5) in the 2050s and 2090s. The results showed that the area of highly suitable zone was highest in the 2090s under scenario SSP5‐8.5 with 8.20 × 10^4^ km^2^, followed by the 2050s under scenario SSP1‐2.6 with 8.05 × 10^4^ km^2^. The total area of suitable zones decreased in all the scenarios compared to the current one, with the highest decrease in the 2050s under scenario SSP1‐2.6, with a decrease of 2.13%. Individually, the 2050s SSP2‐4.5 scenario had the largest decrease in the area of low suitability zones, with a decrease of 42.66%, followed by the 2090s SSP2‐4.5 scenario with a decrease of 18.08%. The low suitability zone area only showed an increase in the 2050s SSP5‐8.5 scenario compared to the current one, with an increase of 3.55%. The change in the area of medium suitability zone is the opposite of the change in the area of low suitability zone, which only decreased by 1.51% in the 2050s SSP5‐8.5 scenario and increased in the rest of the period. The highest increase in the area of the medium suitable zone was observed in the 2050s under the SSP2‐4.5 scenario with an increase of 19.97%, followed by an increase of 11.36% in the 2090s under the SSP2‐4.5 scenario. There was a substantial decrease in the area of the high suitability zone, especially under the 2050s SSP5‐8.5 scenario and the 2090s SSP2‐4.5 scenario, with the largest decreases of 46.44% and 36%, respectively. The area of highly suitable areas shows an increase under scenarios SSP5‐8.5 in the 2090s and SSP1‐2.6 in the 2050s, with increases of 6.95% and 5.21%, respectively (Table 2).

**TABLE 2:** Predicted area of *Graphium sarpedon* in future period (2050s and 2090s and its comparison with the current distribution).

Period	Scenarios	Predicted area (10^4^ km^2^)				Comparison with current distribution (%)			
		Low	Medium	Highly	Total	Low	Medium	Highly	Total
Current	–	97.70	101.18	7.63	206.52	–	–	–	–
2050s	SSP1‐2.6	83.64	110.51	8.05	202.20	−16.81	8.44	5.21	−2.13
	SSP2‐4.5	68.48	126.44	7.54	202.46	−42.66	19.97	−1.19	−2.01
	SSP5‐8.5	101.30	99.67	5.21	206.18	3.55	−1.51	−46.44	−0.16
2090s	SSP1‐2.6	95.64	102.12	5.61	203.37	−2.15	0.92	−36	−1.54
	SSP2‐4.5	82.74	114.15	7.02	203.91	−18.08	11.36	−8.68	−1.28
	SSP5‐8.5	89.41	105.36	8.20	202.97	−9.27	3.96	6.95	−1.75

It can also be seen from Figure 2 that the increase or decrease in the area of highly suitable areas in different periods is mainly concentrated in Guangxi and Guangdong provinces. The increase in the area of medium suitable areas and the decrease in the area of low suitable areas are mainly due to the fact that the low suitable areas in Hubei, Hunan, and Jiangxi provinces have become medium suitable areas.

### 3.4 Environmental variables influencing the distribution

The key environment variables identified through the VIF values were bio8, bio9, bio15, bio18, bio19, prec10, prec4, prec5, prec6, prec9, and aspect (Table S2). The regularized training gain of each environment variable was calculated by jackknife (Figure S2), in which the regularized training gain of bio18, prec4, prec5, prec6, and prec9 exceeded 0.9, indicating that these environment variables are the most important ones among the key environment variables. The other key environment variables bio8, bio9, bio19, and prec10 have regularized training gains above 0.8, indicating that they also play an important role.

**FIGURE 3:**
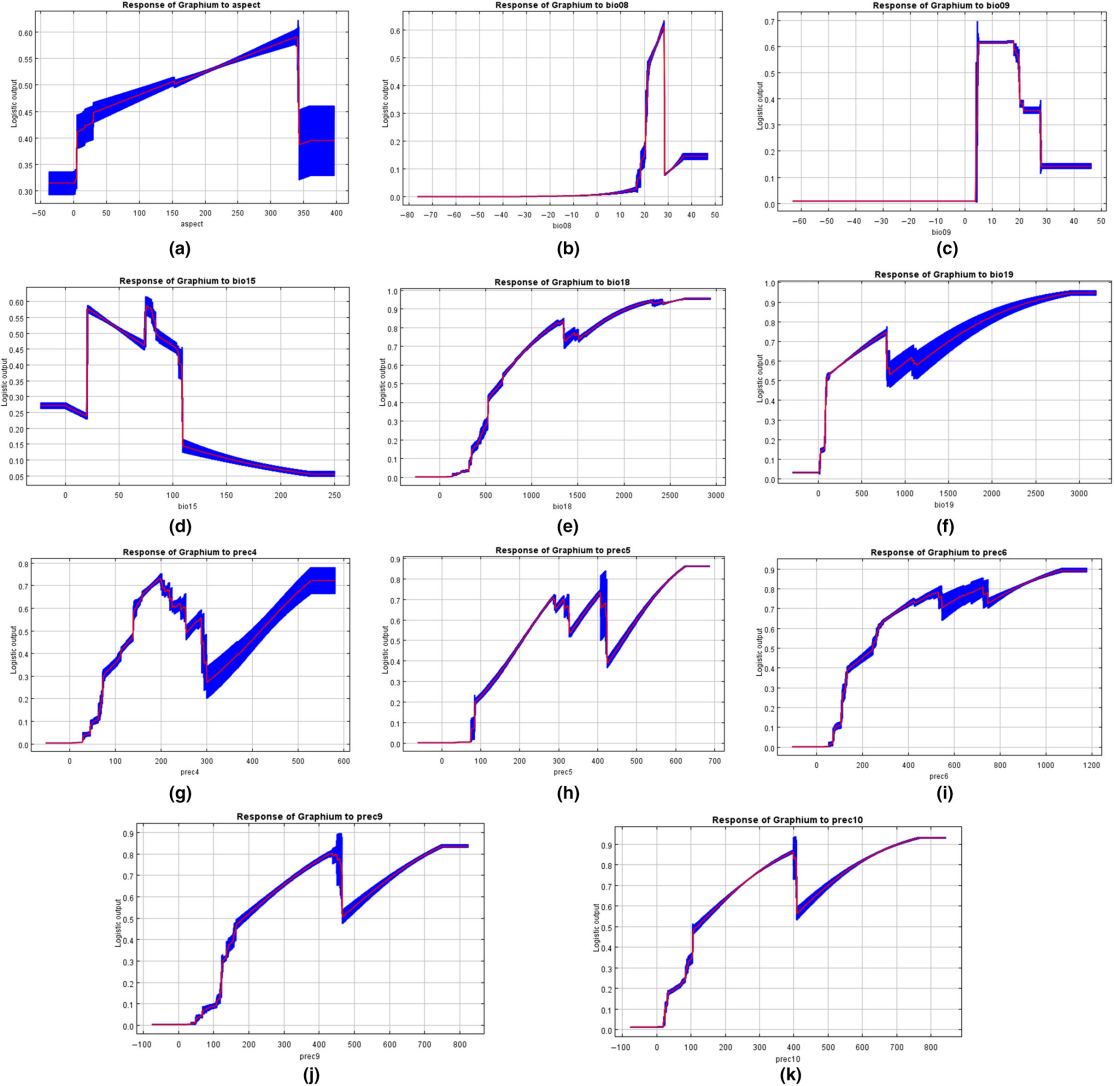
Response curve of dominant environmental variables to occurrence probability of *Graphium sarpedon*, (a) aspect; (b) bio08; (c) bio09; (d) bio15; (e) bio18; (f) bio19; (g) prec4; (h) prec5; (i) prec6; (j) prec9; (k) prec10.

The range of variation in key environmental variables was analyzed by response curves (presence probability: *p* > .66) (Figure 3). Presence probability of *G. sarpedon* was maximum when aspect was 348.98°. Mean Temperature of Wettest Quarter (bio8) at 20.71°C presence probability of *G. sarpedon* is maximum. Mean Temperature of Driest Quarter (bio9) was 18.93°C at which presence probability was maximum. Precipitation Seasonality (Coefficient of Variation) (bio15) was maximum at 83.89 mm. Precipitation of Warmest Quarter (bio18) >910.54 mm was the optimal survival range for *G. sarpedon*, and the presence probability was greatest at 2623.66 mm. Precipitation of Coldest Quarter (bio19) at 499.42–750.77 and >1359.01 mm is the optimum range for survival of *G. sarpedon*, with the highest probability of presence at 2870.77 mm. April precipitation (prec4) was optimal for *G. sarpedon* at 156.79–220.58 and >482.64 mm, with the highest probability of presence at 584.22 mm. The suitable range of May precipitation (prec5) is complex, with 252.92–325.55, 370.74–425.01, and >509.4 mm being the optimal range for *G. sarpedon* to survive, and the probability of presence is maximized at 680.29 mm. June precipitation (prec6) >321.87 mm were all optimal survival ranges for *G. sarpedon*, and the probability of presence was greatest at 1189.92 mm. September precipitation (prec9) at 226.92–470.24 and >569.44 mm was the optimal range for *G. sarpedon* survival, and the probability of presence was greatest at 828.91 mm. October precipitation (prec10) at 211.28–402.49 and >470.37 mm was the optimal range for *G. sarpedon* survival, and the probability of presence was greatest at 826.85 mm. These findings suggest that temperature and precipitation are the primary environmental factors influencing the distribution of *G. sarpedon*.

## 4 DISCUSSION

Species distribution models (SDMs) have been commonly used to predict the response of species to climate change (Laeseke et al., 2020). The MaxEnt model is a reliable software for predicting the suitable area of a species based on the theory of maximum entropy. By simulating and analyzing the distribution state of a species when the entropy reaches a maximum under restricted conditions, it can provide a prediction of potential occurrence areas based on known information and corresponding environmental variables (Wang et al., 2019). In this study, the MaxEnt model and ArcGIS software were used to predict the current and future potential distributions of *G. sarpedon*. The AUC value obtained in this study was 0.977, indicating that the prediction results were highly reliable and could be used as a reference for the potential geographic distribution of *G. sarpedon*.

In this study, the current distribution of *G. sarpedon* is mainly in the plains of the middle and lower reaches of the Yangtze River, the Pearl River Delta, the Yangtze River Delta, and the Lingnan region, and also in the plains of Sichuan and the vast majority of Yunnan. In previous studies, *G. sarpedon* was mainly distributed in Sichuan, Jiangsu, Zhejiang, Jiangxi, Guangdong, Guangxi, Guizhou, and Yunnan provinces, which is consistent with our findings (Wang & Liu, 2009). The primary host plant for *G. sarpedon* is *C. camphora*. *C. camphora* is mainly native to southern and southwestern provinces of China, growing south of the Yangtze River and in parts of southwestern China, which coincides with the current suitability area of *G. sarpedon*. The response curves showed that Mean Temperature of Wettest Quarter (bio8) at 20.71°C presence probability of *G. sarpedon* is maximum, and mean Temperature of Driest Quarter (bio9) was 18.93°C at which presence probability was maximum. The precipitation with the highest probability of presence of *G. sarpedon* in the coldest and warmest seasons was 2870.77 and 2623.66 mm, respectively. The average annual temperature in the subtropical region is 15–20°C, the average temperature of the hottest month is generally higher than 22°C, and most of the annual precipitation is between 800 and 1600 mm, which is not in the optimal range, which can also explain why the middle and lower Yangtze River plains in this work are the current medium suitable and low suitable areas for *G. sarpedon*. The tropical monsoon climate is characterized by year‐round temperatures ranging from 16 to 35°C and annual precipitation ranging from 1500 to 2000 mm. Guangdong and Taiwan are among the most suitable areas for the distribution of *G. sarpedon*, as they are all parts of the tropics (Wu et al., 2000). In the future, global warming will cause the subtropical monsoon climate boundary to move northward, which suggests that *G. sarpedon* is adapting to the new climate change by migrating to higher latitudes. The global climate will continue to warm in the future, and in this context, the precipitation in the middle and lower reaches of the Yangtze River will also increase (Kong, 2020), which will be more favorable to the survival of *G. sarpedon*, and this may also be the reason for the increase of suitable area in the future in the areas of Hubei, Hunan, and Anhui.

Climate is the most critical factor that determines the distribution of species (Castex et al., 2018). Climate change has the ability to directly impact the core life activities of insects, and butterflies can respond quickly to changes in climate, humidity, temperature, and other environmental factors (Soule et al., 2020). Temperature and precipitation are crucial environmental factors affecting the diapause of butterflies (Ji, 2011). This study also found that temperature and precipitation are also key environmental variables that affect the distribution of *G. sarpedon*.

Rainfall plays a crucial role in the growth and reproduction of insects by affecting the humidity of their environment (Xu et al., 2022). Butterflies, in particular, have a high requirement for humidity during their larval stage (Gupta et al., 2019). Humidity affects larval pupation; for example, larvae of *Ostrinia furnacalis* (Guenée) of Lepidoptera showed a consequent shortening and significant difference in the progress of pupation and plumage as the relative humidity of the environment increased (Ding et al., 2022). Adequate humidity levels (85%–90%) are favorable for their reproduction and activities, and this is consistent with the finding that bio15, bio18, bio19, and prec are among the key environmental variables influencing the distribution of *G. sarpedon*. April through May is the period when *G. sarpedon* plumages into adults (Zhang, 2016), and this study also found that precipitation in April and May is a key environmental variable that affects its distribution. Temperature is also a critical factor affecting species distribution (Thomas et al., 2004). Butterflies are poikilothermic animals that are highly sensitive to changes in their external environment. Temperature increases have a direct impact on their biological habitat, causing changes in other environmental factors and triggering their redistribution (Hughes, 2000). Bio8 and bio9 are considered a crucial environmental variable influencing the distribution of *G. sarpedon*. Previous research has found that the optimal temperature for *G. sarpedon* larvae development is between 20 and 28°C, and the ideal temperature range for adults is 18–30°C, which is consistent with the results of this study (Figure S3D) (Wu, 2018). This implies that as temperatures continue to rise, the activity period of *G. sarpedon* may occur earlier, which could make it challenging for these butterflies to find a suitable environment in time. In the future, changes in temperature and precipitation could be the primary factors contributing to a reduction in the suitable habitat for these butterflies.

One of the key contributions of our study is the use of the MaxEnt model to determine the distribution probability of *G. sarpedon* in China, which will effectively guide future field investigations, monitoring, and control efforts. While the MaxEnt model has the advantages of being easy to use, requiring small sample sizes, and providing high prediction accuracy, it also has some limitations; for example, response curves show only the effect of a single environmental factor, ignoring interactions between variables (Deng et al., 2022). In this study, we focused on the impact of climate on the distribution of *G. sarpedon*, and the environmental variables used in the MaxEnt model prediction, with the exception of altitude, were all climate variables. However, in reality, biological factors, such as species interactions, vegetation types, geomorphological features, the species' own dispersal ability, the presence of natural enemies, and human activities, may also have a significant impact on the potential distribution of the predicted species. Therefore, while there may be some differences between the distribution predicted by the MaxEnt model and the actual distribution of *G. sarpedon*, our prediction of their distribution based on climatic factors would be still reliable and can improve the efficiency of quarantine and control measures in high distribution areas. However, there are many shortcomings. The distribution of *G. sarpedon* can also be influenced by its host plants. As an oligophagous insect, *G. sarpedon* relies heavily on Lauraceae plants for its life activities (Xiao, 2020). However, temperature can play a critical role in the physiological and ecological processes of plants (Hu et al., 2021). Photosynthesis, in particular, is a physiological phenomenon that plants are highly sensitive to, and it can directly affect the growth and development of plants (Calzadilla et al., 2022). High temperatures can stress plants, damage their photosynthesis, and reduce their photosynthetic rate. These effects of climate change on Lauraceae plants could, in turn, have cascading effects on the growth and distribution of *G. sarpedon*. This work does not consider the effect of host plants on the distribution of *G. sarpedon*. To improve future research, we should consider various environmental factors, including biological factors, and compare the impact of different types of models on the prediction results to improve accuracy and prediction effectiveness. Additionally, the environmental variables selected for the forecast were based on data only from 1970 to 2000, and the recent 20 years of climate data are missing. Therefore, in the future, we should ensure that the missing data are accounted for to make our prediction results more prepared and reliable.

## 5 CONCLUSION

In this study, we predicted the current and future potential distribution of *G. sarpedon* in China based on the MaxEnt model and identified key environmental variables affecting its distribution. The results showed that under current conditions, the highly suitable areas for *G. sarpedon* are mainly distributed in the provinces of Guangdong, Guangxi, Hainan, Taiwan, and Hong Kong. Under future conditions, the total suitable area decreased, although some areas in Hubei, Hunan, and Anhui provinces changed from low to high suitable areas. The most critical environmental variables affecting the distribution of G. sarpedon are temperature and precipitation. This study facilitates early detection and prevention to minimize the ecological damage caused by *G. sarpedon*.

## AUTHOR CONTRIBUTIONS

**Wenkai Liao:** Investigation (equal); methodology (equal); software (equal); writing – original draft (equal). **Zhihang Zhuo:** Investigation (equal); methodology (equal); writing – review and editing (equal). **Qianqian Qian:** Investigation (equal); software (equal); visualization (equal). **Dan Hu:** Software (equal); writing – review and editing (equal). **Danping Xu:** Conceptualization (equal); formal analysis (equal); supervision (equal).

## FUNDING INFORMATION

This research was funded by the Ministry of Science and Technology of the People’s Republic of China Support Program (2022YFE0115200), Sichuan Province Science and Technology (2022NSFSC0986), and China West Normal University (20A007, 20E051, 21E040, and 22kA011).

## CONFLICT OF INTEREST STATEMENT

The authors declare that they do not have any kind of conflict of interests.

## Supporting information


Appendix S1.

## Data Availability

The data supporting the results are available in a public repository at: GBIF.org (May 11, 2022) GBIF Occurrence Download https://doi.org/10.15468/dl.mzhk5g. Longitude and latitude coordinates of *G. sarpedon*. figshare. Dataset. https://doi.org/10.6084/m9.figshare.24624066.v1.
