# Supplementary material for: Potential impact of climatic factors on the distribution of Graphium sarpedon in China
Source: Ecol Evol. 2024 Feb 6;14(2):e10858. doi: 10.1002/ece3.10858 (PMC10847806; doi:10.1002/ece3.10858)
Supplement: Supplementary file 1 — Appendix S1. [file ECE3-14-e10858-s001.docx]

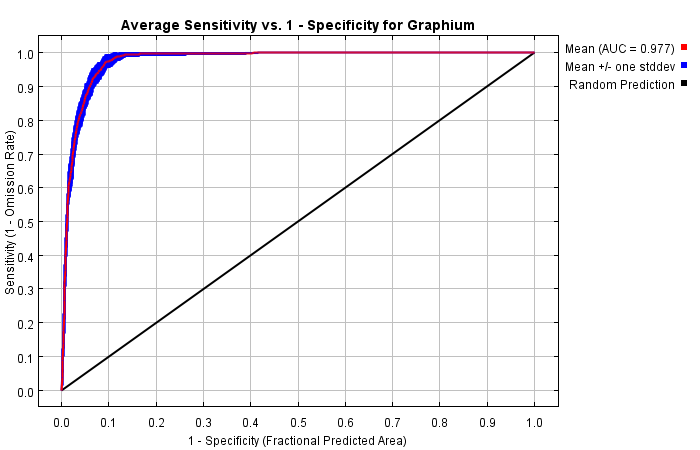


Fig S1 AUC result of MaxEnt modelling


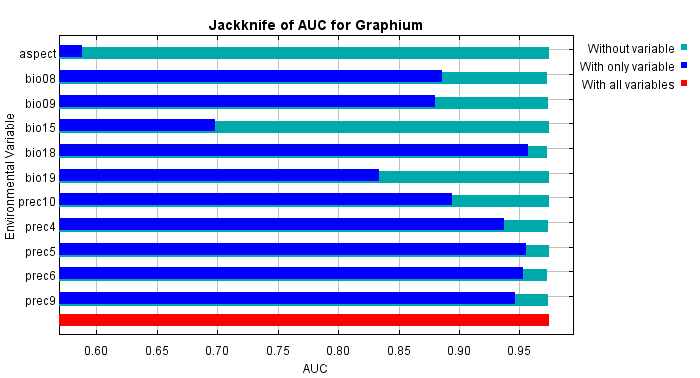


Fig S2 The inportance of environment variables to G.sarpedon by jackknife test

| **Table S1**  The evaluation criteria of modeling performance. | |
| --- | --- |
| Value of AUC | Evaluation Criterion |
| 0.5 | Fail |
| 0.6≤AUC＜0.7 | Barely available |
| 0.7≤AUC＜0.8 | Acceptable |
| 0.8≤AUC＜0.9 | Good |
| 0.9≤AUC＜1.0 | Excellent |

| **Table S2** | | |
| --- | --- | --- |
| Vif value of each environmental variables. | | |
| Environment variables | Significance | Vif value |
| aspect | 0.991 | 1.650 |
| bio15 | 0.132 | 81.583 |
| bio18 | 0.000 | 33.164 |
| bio19 | 0.002 | 9.769 |
| bio8 | 0.000 | 23.542 |
| bio9 | 0.467 | 85.116 |
| prec 04 | 0.670 | 54.017 |
| prec 05 | 0.099 | 40.073 |
| prec 06 | 0.483 | 71.044 |
| prec 08 | 0.442 | 80.585 |
| prec 09 | 0.143 | 44.203 |
| prec 10 | 0.066 | 68.169 |
| prec 11 | 0.001 | 95.246 |

| **Table S3**  Predicted area of *G. sarpedon* in current period. | | | | | |
| --- | --- | --- | --- | --- | --- |
| Province | Area (10^4^ km^2^) | | | Suitable area ratio (%) **^a^** | |
|  | Low suitable area | Medium suitable area | Highly suitable area | |  |
| Gansu | 0.021 | 0.00 | 0.00 | | 0.04 |
| Jilin | 0.24 | 0.00 | 0.00 | | 1.12 |
| Tibet | 0.90 | 2.75 | 0.99 | | 4.06 |
| Shaanxi | 4.20 | 0.057 | 0.00 | | 20.94 |
| Liaoning | 0.16 | 0.00 | 0.00 | | 1.02 |
| Henan | 5.14 | 0.014 | 0.00 | | 31.94 |
| Sichuan | 5.15 | 12.07 | 0.00 | | 37.62 |
| Yunnan | 14.11 | 8.94 | 0.00 | | 67.33 |
| Hong Kong | 0.00 | 0.0017 | 0.085 | | 100 |
| Hainan | 0.49 | 2.40 | 0.00 | | 99.82 |
| Guangdong | 0.82 | 10.97 | 3.81 | | 1 |
| Guangxi | 1.05 | 18.91 | 0.96 | | 99.99 |
| Taiwan | 0.05 | 1.57 | 1.57 | | 1 |
| Fujian | 4.48 | 6.5 | 0.003 | | 1 |
| Jiangsu | 3.81 | 0.68 | 0.00 | | 46.72 |
| Zhejiang | 0.41 | 8.68 | 0.26 | | 1 |
| Guizhou | 10.79 | 4.48 | 0.00 | | 95.61 |
| Jiangxi | 9.72 | 5.53 | 0.00 | | 1 |
| Hunan | 16.27 | 3.08 | 0.00 | | 1 |
| Shanghai | 0.21 | 0.38 | 0.00 | | 1 |
| Anhui | 4.38 | 4.31 | 0.00 | | 64.99 |
| Chongqing | 2.02 | 5.69 | 0.00 | | 1 |
| Hubei | 13.33 | 4.23 | 0.00 | | 99.88 |
| Total area | 97.75 | 101.24 | 7.68 | | 43.63 |
| **^a^** Means the ratio of suitable area (highly + medium + low suitable area) to the corresponding province’s total land area. | | | | | |
